# The Potential Use of Intrauterine Insemination as a Basic Option for Infertility: A Review for Technology-Limited Medical Settings

**DOI:** 10.1155/2009/584837

**Published:** 2009-04-14

**Authors:** Abdelrahman M. Abdelkader, John Yeh

**Affiliations:** Department of Gynecology-Obstetrics, University at Buffalo, The State University of New York, Buffalo, NY 14222, USA

## Abstract

*Objective*. There is an asymmetric allocation of technology and other resources for infertility services. Intrauterine insemination (IUI) is a process of placing washed spermatozoa transcervically into the uterine cavity for treatment of infertility. This is a review of literature for the potential use of IUI as a basic infertility treatment in technology-limited settings. *Study design*. Review of articles on treatment of infertility using IUI. *Results*. Aspects regarding the use of IUI are reviewed, including ovarian stimulation, semen parameters associated with good outcomes, methods of sperm preparation, timing of IUI, and number of inseminations. Implications of the finding in light of the needs of low-technology medical settings are summarized. *Conclusion*. The reviewed evidence suggests that IUI is less expensive, less invasive, and comparably effective for selected patients as a first-line treatment for couples with unexplained or male factor infertility. Those couples may be offered three to six IUI cycles in technology-limited settings.

## 1. Introduction

Intrauterine insemination (IUI) is the therapeutic process of placing washed spermatozoa transcervically into the uterine cavity for the treatment of infertility. IUI theoretically allows a relatively higher number of motile spermatozoa to reach the oocyte [1]. The rationale for washing sperm is to remove prostaglandins, infectious agents, and antigenic proteins as well as to remove immotile spermatozoa, leucocytes, and immature germ cells. The process allows the concentration of spermatozoa in a small volume of culture media and then the concentrated spermatozoa is placed into the uterus through a transcervical catheter.

The general process of artificial insemination has been used to treat a variety of physiological and psychological male and female infertility disorders such as severe hypospadius, retrograde ejaculation, impotence, and vaginismus. IUI, in the past, has been used as treatment for poor postcoital tests and immunologic infertility [2]. Currently, IUI is used to treat moderate male factor infertility and unexplained infertility. Another common use of IUI is to enhance the efficacy of treatment by ovulation induction for ovulatory disorders [1]. The simple and noninvasive nature of IUI has allowed it to be performed by nurses in some centers with analogous pregnancy rates to the procedures performed by physicians [3]. Factors that may influence IUI outcome include the use of ovulation induction agents, semen analysis parameters, techniques used for sperm preparation, and the timing and number of inseminations.

The resource allocation for advanced infertility services is asymmetric, with the expertise and technology more concentrated in the larger cities and resource-rich westernized countries [4]. In this review, we address the clinical situation in the majority of the occasions, in which the infertility technology that is available for treatment of patients may be limited. Outside the technologically advanced centers, the majority of the infertility patients have fewer options for treatment. The roles for IUI and considerations for its advantageous use in this limited technology setting are considered in this manuscript. This review is divided into two parts. In the first, the efficacy and cost-effectiveness of IUI are discussed, and, in the second part, the evidence is presented for the beneficial clinical practice of IUI in limited technology settings.

## 2. Infertility and Its Economic and Psychological Impacts

Infertility is defined as failure to achieve pregnancy for one year or more without use of contraception during the childbearing period and affects approximately 10–15% of couples [5]. In 1995, The National Survey of Family Growth reported that 9.3 million women between 15–44 years (15% of women of reproductive age) made use of infertility services in the United States. These services included medical advice, testing for both couples, drugs for ovulation induction, corrective surgery for tubal blockage, and assisted reproductive technologies (ARTs) [6]. The costs of infertility evaluation and therapy were evaluated in a study conducted by Stovall et al. [7]. They found that the costs of infertility services ranged from 0.36 to 1.03% of a total health care plan with an average of 0.8%. In a 3-year period, they found that infertility cost was $680 921 of $86 445 642 total health care plan costs.

In addition to its economic costs, infertility has a major psychological impact. Oddens et al. reported that infertile women had depressive and anxiety symptoms four times more frequently than fertile women [8], and the rate is comparable to women with cancer and coronary heart disease [9]. The presence of such psychological disorders may have a negative impact on infertility treatment outcomes, as it was reported that IVF success rates in the first 5 cycles may be lower in depressed women compared to the nondepressed [10].

## 3. Limitations of Assisted Reproductive Technologies

In 2001 in the United States, 108 130 ART cycles were carried out in 385 programs, with 79 042 cycles being IVF [11]. In 2003 in Europe, 365 103 ART cycles were accomplished in 1008 clinics, with 132 932 cycles being IVF [12]. Despite this increase in the use of these treatment modalities, they are still costly, invasive, and associated with drawbacks. In the year 2001, the median cost of IVF in the US was extrapolated to be $9 226 per cycle and $56 419 per delivery. Outside the US, this cost was calculated to be $3 531 per cycle and $20 522 per delivery [13]. The high cost of IVF deepens on the gap between the number of IVF cycles performed and the treatment needs of infertile couples.

In addition to its costs, the use of IVF is associated with other undesirable outcomes. These include preterm delivery, leading to increased risk of newborn prematurity and its concomitant costs and morbidity, and increased rates of multiple gestations [14]. In the US, in the year 2003, 48 756 infants were born from ARTs, accounting for 1% of all births. Fifty-one percent of this number resulted from multiple gestations. Furthermore, the incidence of low birth weight and preterm delivery among singleton infants born from ARTs were 9% and 15%, respectively, while low birth weight occurred in 94% among triplets and higher order multiple gestations [15]. In a meta-analysis to assess the perinatal outcome in singletons following IVF [16], Jackson et al. reported increased fetal risks of preterm birth, low and very low birth weights, small for gestational age, neonatal intensive care unit admissions, stillbirth, neonatal mortality, and cerebral palsy as well as increased maternal risks of pre-eclampsia, placenta previa, gestational diabetes, and caesarean delivery. Furthermore, there may be an increased incidence of major birth defects (renal and musculoskeletal) and autosomal reciprocal-balanced translocations among infants born after IVF/ICSI compared to naturally conceived infants [17, 18].

## 4. Limitations of IUI

The limitations of IUI are few. They included the potential risk of infection from uterine catheterization and injection of the semen specimen. However, this risk has been reported to be 0.01–0.2%. In addition, ectopic pregnancies and spontaneous miscarriages may occur, which is not different from other infertility treatment modalities [19]. The production of antisperm antibodies is another hypothetical drawback of the use of IUI. The incidence of multiple pregnancy and ovarian hyperstimulation syndrome (OHSS) with IUI may occur and is related to the use of ovarian stimulation with IUI. The risk of OHSS may be decreased by use of low-dose drug regimens, close monitoring, and strict cycle cancellation criteria.

Regarding the perinatal outcomes of IUI conceptions, Gaudoin et al. reported in a retrospective cohort study that ovulation induction combined with IUI was associated with increased risk of preterm birth and low birth weight [20]. However, other studies did not describe such associations [21, 22].

## 5. Effectiveness of IUI

The pregnancy rate of IUI is reported to 10–20% per patient, but the reported rates range from as low as 5% to as high as 70% [23]. Based on the etiology of infertility, the highest rates were reported when IUI was used in patients with anovulation who were undergoing ovulation induction therapy at the time of the IUI treatment, male factor infertility, and unexplained infertility. In patients with endometriosis, the pregnancy rates were the lowest [24]. The number of mature follicles (17 mm in diameter or more) is another prognostic factor in IUI success, where the presence of 3-4 mature follicles was associated with higher pregnancy rates and a lower incidence of high-order multiple pregnancies [25]. Other prognostic factors included female age, infertility duration and amount of motile sperm [26].

The cost effectiveness of IUI has been studied. In a retrospective cohort study [27], Van Voorhis et al. reported $8 674 as the cost per delivery for IUI alone, $7 808 for clomiphene combined with IUI, $10 282 for human menopausal gonadotropin (hMG) combined with IUI and $43 138 for IVF. The authors supported the use of IUI (any modality) as being cost effective, before ART, for infertile couples with patent tubes, female age 38 years or less, and a sperm count of 10 × 10^6^ motile spermatozoa in a post wash sample. For those with blocked tubes, IVF-ET was found to be a cost-effective practice compared to surgery. In a prospective study, based on cost effectiveness, Karande et al. did not recommend IVF as first-line option for infertile couples [28]. In a prospective-randomized study, Goverde et al. found that IUI was as effective as and less costly than IVF in treatment of unexplained and male factor infertility [29]. Peterson et al. reported that four cycles of controlled ovarian hyperstimulation (COH) combined with IUI were superior to IVF and less expensive than single IVF cycle [30]. Even when the same stimulation protocols were used, the cost per pregnancy for IUI was less than half that of IVF [31]. Cohlen et al. conducted a Cochrane review to analyze the effectiveness of IUI versus timed intercourse both in natural and stimulated cycles in cases of male factor infertility [32]. A search of Medline (January 1966-present), EMBASE (http://www.embase.com/), DDFU ( http://www.ovid.com/site/catalog/DataBase/893.jsp?top=2&mid=3&bottom=7&subsection=10), BIOSIS (Philadelphia, USA), SCI ( http://www.thomsonreuters.com/products_services/scientific/Science_Citation_Index_Expanded) and hand searching of references from identified studies resulted in 43 studies related to research topic being retrieved. They used 17 studies in the systematic review and their data were pooled in the meta-analysis. In their final conclusions, Cohlen et al. reported that IUI with COH was more cost-effective compared to IVF [32]. A more recent study, by Pashayan et al., however, offered evidence that IVF as a first-line treatment for couples with unexplained and mild male factor infertility was less costly than IUI followed by IVF (for IUI treatment inability to achieve pregnancy) [33].

## 6. The Use of IUI and Concurrent Ovarian Stimulation

Which is more effective: IUI in a natural cycle or combined with ovarian stimulation? In a meta-analysis that included only randomized controlled trials, Hughes reported a significant improvement in fecundity with the use of IUI and follicle stimulating hormone (FSH). He found more than two-fold increase in fecundability for either IUI or FSH treatment alone, while he found a five-fold increase when IUI and FSH are combined together [34]. In the Cochrane review by Cohlen et al. referenced above, conducted to assess the effectiveness of IUI versus timed intercourse [32], IUI in a natural cycle was compared to IUI combined with controlled ovarian hyperstimulation. Data from four trials were included in the meta-analysis for this comparison. Although there was no significant difference between IUI in natural cycles and IUI combined with ovarian hyperstimulation in cases with male factor infertility, the results suggested increased conception with ovarian hyperstimulation (odds ratio: 1.8 with CI: 0.98–3.3). In their conclusion, Cohlen et al. recommended the use of IUI in natural cycles for cases with severe semen defect (a total motile sperm count of less than 10 × 10^6^, but with more than 1 × 10^6^ motile sperm after preparation). In cases of less severe semen defect, IUI with ovarian hyperstimulation was recommended. A large randomized study in the US confirmed the effectiveness of combining ovarian stimulation with IUI [35]. The study reported an overall pregnancy rate per couple of 33% for IUI with ovarian stimulation and an overall pregnancy rate of 18% for IUI alone. Data of this study were included in the Cochrane review, discussed above, which was accomplished to assess the effectiveness of IUI in the treatment of unexplained infertility. Verhulst et al. conducted a Cochrane review to assess the effectiveness of IUI in the treatment of unexplained infertility [36]. Their primary search yielded 198 articles related to the topic, but hand searching of their abstracts resulted in 25 trials included in the review. It was found that live birth rate per couple was significantly higher when IUI was combined with ovarian hyperstimulation compared to IUI alone (OR 2.07, 95% CI: 1.22–3.5) [36]. However, this review did not find studies assessing expectant management versus IUI in unexplained infertility. At the same year of publication as the above Cochrane review, Steures et al. published a randomized clinical trial comparing ovarian hyperstimulation combined with IUI versus expectant management for couples with unexplained infertility [37]. This study reported no higher pregnancy rates for ovarian hyperstimulation combined with IUI overexpectant management in couples with unexplained infertility.

The National Institute for Clinical Excellence (NICE) in the United Kingdom recommended, with level I evidence, that when IUI is used to treat male factor infertility, ovarian stimulation should not be offered. This conclusion was based on two trials [29, 32]. Supporting this recommendation is the results of a randomized clinical trial conducted in the Netherlands by Steures et al. [38]. This study reported similar pregnancy rates when IUI alone or IUI combined with ovarian hyperstimulation were used to treat couples with abnormal postcoital tests and poor prognosis for spontaneous pregnancy. For unexplained infertility, both IUI alone and IUI combined with ovarian hyperstimulation appear to be more effective than expectant management alone [39].

What drugs should be used for ovarian hyperstimulation combined with IUI? Gonadotropins with and without gonadotropin-releasing hormone (GnRH) agonist/antagonist have been studied, along with recombinant human FSH (rFSH). Ragni et al. reported that the daily use of 50 IU of rFSH, combined with a GnRH antagonist, ganirelix, for ovarian stimulation prior to IUI resulted in a live birth rate per couple of 25.7% [40]. When the same dose of rFSH was used on alternate days, rFSH produced a live birth rate of 2.9% [40]. In another study, 50 IU daily of rFSH was found similar to 75 IU daily of urinary FSH (uFSH) in the outcomes of the number of follicles >17 mm and days of stimulation [41]. The clinical pregnancy rates were 12.7% for rFSH and 11.9% for uFSH [41]. Thus, the more expensive rFSH was not a cost-effective drug for induction of ovulation in IUI cycles. Conversely, Demirol and Gurgan compared rFSH to uFSH and hMG in IUI cycles, and found rFSH and IUI produced a clinical pregnancy rate of 25.9% compared to 13.8% for uFSH and 12.5% for hMG [42]. When rFSH and clomiphene citrate (CC) were compared for ovulation induction with IUI in couples with unexplained and male factor infertility, the cumulative pregnancy rate was 38% for CC-IUI group versus 34.3% for the rFSH-IUI group [43].

Recently Cantineau and Cohlen performed a Cochrane review to evaluate different ovarian stimulation protocols (antiestrogens, aromatase inhibitors, and gonadotropins with or without GnRH agonists/antagonists) [44]. All published randomized controlled trials (RCTs) comparing different stimulation protocols before IUI were searched in the Menstrual Disorders and Subfertility group's Central register of the Controlled Trials, Medline, and EMBASE. 81 studies were identified, but only 43 trials met the inclusion criteria. Of the different comparisons done in this review, letrozole was not more effective than CC (OR 1.2, 95% CI: 0.64–2.1). The analysis also revealed that gonadotropins, in low-dose regimens (50–75 IU), were the most effective agents when ovarian stimulation was combined with IUI. Although less effective than gonadotropins, antiestrogens were more cost effective in IUI therapy. Neither a higher dosage (>75 IU gonadotropins) nor the addition of GnRH agonists was more effective. Conversely, the higher dosage and the GnRH agonists were associated with increased costs and risks of multiple gestations and of OHSS. A systematic review by Costello assessed the effectiveness of CC and IUI in the treatment of ovulatory infertility [45]. Seven RCTs were included in this review. The meta-analysis of this review showed a cycle pregnancy rate of 14.3% for CC combined with IUI versus 6.4% for natural cycle IUI.

**Considerations in Technology-Limited Settings**

The evidence reviewed suggests that oral clomiphene therapy combined with IUI or natural cycle IUI is satisfactory first-line choices for treatment of unexplained and male infertility in low-technology settings. The combination of IUI and controlled ovarian hyperstimulation may result in a relatively higher therapeutic pregnancy rate for unexplained and for male infertility. Gonadotropins are the controlled ovarian hyperstimulation agents that result in the highest rates of pregnancy. However, the costs associated with these drugs are higher, and the serum steroid measurements and ultrasonographic monitoring require higher levels of technological capabilities.

## 7. Semen Parameters and the Use of IUI

The WHO criteria for normal semen parameters are widely used [46]. In the system, an abnormal semen count is defined as deviations from the normal criteria on two consecutive semen analyses [46]. Studies have examined semen parameters, like motile sperm concentration and normal sperm morphology, in relation to IUI outcome (Table 1). In Table 1, four inferences could be made: most of the studies are retrospective, the number of insemination cycles is fewer in the prospective studies, all but one study used the inseminated motile sperm count after sperm preparation and the studies reported on different outcome measurements. In spite of the limitations outlined above, it may be deduced that reasonable IUI success rates can still be obtained in cases of severe semen defects (normal morphology 4% or less and inseminated sperm count as low as one million). This supports the need for large well-designed prospective studies using standard outcomes.

Regarding sperm concentration, Ombelet et al. in a retrospective study found that when inseminated motile sperm count was >1 × 10^6^, clomiphene-IUI resulted in a baby take-home rate of 21–25% after 3 cycles [47]. Even when inseminated motile sperm count was < 1 × 10^6^ and the normal sperm morphology >4%, clomiphene-IUI remained a treatment choice that resulted in pregnancies. In a retrospective analysis of 3 479 IUI cycles conducted for 1039 infertile couples [48], Van Voorhis et al. found that a total motile sperm count per specimen of less than 10 million was associated with lower pregnancy rates. On the other hand, a total motile sperm count above 10 million did not produce a significant increase in IUI pregnancy rates.

Cohlen et al. defined a total motile sperm count per ejaculate between 5 and 10 × 10^6^ to be a severe semen defect [49]. They reported a pregnancy rate per IUI cycle of 12% for couples with that count. Wainer et al. reported on the number of motile sperm inseminated [50] and found a clinical pregnancy rate per cycle of 3.13% when the number of motile sperm inseminated was < 1 × 10^6^. When the number of motile sperm inseminated was between 5 and 10 × 10^6^, the clinical pregnancy rate per cycle was 14%. Miller et al. reported a cutoff point for number of motile sperm inseminated after sperm preparation as 10 × 10^6^ [51]. Berg et al. reported successful pregnancies in patients after controlled ovarian stimulations in IUI preparations with motile sperm counts as low as >0.8 × 10^6^ [52].

Normal sperm morphology as a predictor of IUI success has been examined. After controlling for sperm concentration and motility, Lee et al. found that the normal sperm morphology, using Kruger's strict criteria [53], more strongly predicted IUI outcome [54]. They reported pregnancy rates per cycle of 3.8% in couples with <4% of Kruger's strict criteria normal morphology, 18.5% for Kruger's strict criteria normal morphology of 4–9% and 29.9% when Kruger's strict criteria normal morphology was above 9% [54]. These results were similar to those reported in a study by Hauser et al. [55], where CC-IUI resulted in a pregnancy rates per couple of 11.1% when normal morphology by strict criteria was less than 4%, 36.1% for normal morphology of 4–14% and 50% when normal morphology was above 14%. The link between sperm motility and IUI success was addressed in several studies. Briefly, in these studies, total motile sperm of 30–50% before sperm preparation was found to be associated with positive IUI outcomes [54, 56, 57].

**Considerations in Technology-Limited Settings**

The evidence reviewed suggests that IUI may be offered to couples with male factor infertility, in a low technology setting, if the total motile sperm count is more than 5 million per specimen.

## 8. Sperm Preparation Methods

The aim of semen preparation is to separate motile, morphologically normal spermatozoa from the seminal plasma and from debris such as leucocytes, bacteria, and nonmotile spermatozoa. Table 2 shows studies that addressed the effect of different sperm preparation methods on IUI outcome. As shown in the table, the number of these studies is limited. Sperm wash, swim-up, and density gradient centrifugation are the most commonly used methods. As shown in Table 2, density gradient centrifugation was associated with acceptable IUI outcomes [58–62]. Swim-up as a procedure is also associated with satisfactory IUI outcomes, comparable to density gradient centrifugation pregnancy rates. Furthermore, other studies (the preparation technique was not the primary question addressed) reported similar outcomes for both methods [47, 50]. Boomsma et al. conducted a Cochrane review to compare the effectiveness of the three different semen preparation techniques (conventional wash, swim-up procedure, and density gradient centrifugation) before IUI [63]. Only two RCTs could be included in the meta-analysis regarding the clinical outcomes. This resulted in insufficient data to recommend anyone of the three semen preparation techniques over the others.

The time intervals between semen collection, sperm processing, and insemination may affect IUI outcomes. Yavas and Selub performed a retrospective study where the collection of semen at the clinic was found to be better than at home. Short intervals from semen collection to sperm wash, from sperm wash to IUI, and from semen collection to IUI were associated with higher pregnancy rates compared to semen collection at home with longer time intervals [64].

**Considerations in Technology-Limited Settings**

The evidence suggests that conventional sperm wash for IUI can be used in a low-technology setting. The evidence does not seem to show that anyone of the three commonly used sperm preparation methods results in a higher pregnancy rate than any other.

## 9. Timing of IUI

Normal sperm is capable of fertilizing an oocyte in the female genital tract for about 5 days, and an oocyte is fertilizable for 12–24 hours after ovulation [65]. The WHO conducted a large multicenter study that concluded that ovulation occurred between 24 and 56 hours (average 32 hours) after the onset of luteinizig hormone (LH) surge [66]. In another study ovulation, occurred 36–38 hours after the onset of LH surge in natural cycles [67]. The LH surge can be detected at home using urinary LH kits. The monitoring for the urinary LH surge can start about 3 days before the expected date of ovulation. Generally, in natural cycles, IUI can be performed 24 hours after the onset of LH surge as detected by urinary LH monitoring.

In controlled ovarian stimulation cycles in which ovulation is triggered artificially, ovulation occurs 32–38 hours after human chorionic gonadotropin (HCG) injection [68, 69]. Based on the available data about the timing of ovulation, some investigators recommended that insemination can be carried out between 12 and 60 hours after HCG injection [70–72]. Other investigators found that delayed inseminations to 38–40 hours after HCG injection were associated with higher pregnancy rates compared to early inseminations [73]. This was not found in a study by Claman et al., who found that there was no significant difference in pregnancy rates between early (32–34 hours) and late (38–40 hours) inseminations [74]. When HCG is used, insemination can be done 34–40 hours after injection [75].

A systematic review was performed by Kosmas et al. to compare the effectiveness of HCG administration versus urinary LH detection as a method of IUI timing after CC stimulation [76]. Seven studies were included in this systematic review and the meta-analysis. The authors found that the use of urinary LH monitoring as a method of IUI timing was associated with higher pregnancy rates than the HCG administration method. They concluded that LH monitoring for IUI timing is more practical, effective, and cost-effective when CC is used for ovarian stimulation [76].

**Considerations in Technology-Limited Settings**

The evidence suggests that it is reasonable to perform IUI, for either natural cycle IUI or for clomiphene combined with IUI, approximately 24 hours after the detection of the LH surge. Urine testing kits could be used to detect the LH surge.

## 10. Number of Inseminations per Cycle

Several studies have compared single versus double IUIs in an ovulatory cycle. One of the earlier studies, conducted for that purpose, reported a cycle fecundity of 52% when 2 inseminations 18 and 42 hours after HCG were performed while a single insemination performed 34 hours after HCG injection resulted in a cycle fecundity of 8.7% [71]. Another study found that double inseminations, performed 12 and 34 hours after HCG administration, in gonadotropin stimulated cycles, resulted in a pregnancy rate per patient of 30.4%, a higher pregnancy rate than that found from single inseminations (14.4%) performed 34 hours after HCG [72].

On the other hand, Ransom et al. found no difference in pregnancy rates between single IUI performed 35 hours after HCG and double IUI done 19 and 43 hours after HCG injection [77]. These results were supported in a study by Alborzi et al. [78] where pregnancy rates per cycle were 8.6% and 9.4% (nonsignificantly different) for single and double IUIs. Another study, by Casadei et al., failed to show a significant advantage of double inseminations 12 and 36 hours after HCG injection over single insemination done 36 hours after HCG [79].

Two reviews were conducted to reach a conclusion about the number of inseminations per cycle [80]. One was a systematic review and meta-analysis [80] and the second was a Cochrane analysis [81]. In the first review, by Osuna et al., 18 trials were retrieved from searching Medline, The Cochrane library, and the abstract books of annual meetings of ESHRE and ASRM [80]. Only six randomized prospective studies were included in the review. The pooled outcomes of the studies were a pregnancy rate per cycle of 14.9% for double IUIs versus 11.4% for a single IUI. This difference was statistically insignificant. The Cochrane review, by Cantineau et al., followed the same search strategies and data analyses as mentioned previously [81]. From the 30 studies retrieved, 13 were found to provide data comparing single versus double inseminations. Only 3 RCTs were included in the review, the results of 2 of them were pooled for meta-analysis. The authors concluded that there was no evidence that double inseminations give rise to higher live birth rates in infertile couples compared to single inseminations (OR 1.45, CI: 0.78–2.7). The NICE recommendation is for a single insemination when offering IUI as therapy [39].

**Considerations in Technology-Limited Settings**

The evidence suggests that single IUI insemination per ovulatory cycle should be considered. Published evidence does not support that two IUI in a single cycle results in a higher rate of pregnancy. A single insemination will be less costly, with similar pregnancy outcomes.

## 11. Number of Insemination Cycles

Some studies report six treatment cycles [29, 60, 78], while others report four cycles [35, 82] as the number of cycles of treatment with IUIs. One study reported a cumulative probability of pregnancy of 38.2% for 6 treatment cycles and 49.5% for 10 treatment cycles [52]. One of the six-cycle studies, by Morshedi et al., reported that 88% of the pregnancies occurred in the first 3 cycles [60]. However, a Cochrane review and the NICE guidelines support using IUI as treatment for up to 6 cycles [32, 83]. The NICE reported, with level I evidence, that the use of up to 6 cycles on IUI increased the chance of pregnancy for couples with mild male-factor and unexplained infertility and minimal to mild endometriosis. The Cochrane reviewers concluded that most pregnancies occurred during the first 3 to 6 IUIs treatment cycles.

**Considerations in Technology-Limited Settings**

The evidence suggests performing a minimum of three IUI treatment cycles and a maximum of six IUI treatment cycles in technology-limited setting.

## 12. Conclusion

The resource allocation of infertility services available for patients is asymmetric, with the highest levels in the larger cities. The evidence reviewed suggests that IUI may be helpful in a low-technology medical setting. It may be considered as a good first-line treatment for couples with unexplained infertility, male factor infertility, and anovulation (IUI used concurrently with ovulation induction). In the clinical practice of IUI in a low-technology setting, combining oral clomiphene with IUI is as reasonable of an option as natural cycle IUI. For semen parameters, a motile sperm count above 1 million in the final specimen can serve as a cutoff point for offering IUI. Conventional sperm washing, density gradient centrifugation, or swim-up techniques can all be used for sperm preparation before IUI, with conventional sperm washing being the simplest. A single IUI per cycle should be performed, and the IUI can be performed approximately 24 hours after urinary LH surge is detected. Couples may be offered 3 to 6 IUI cycles to ensure sufficient opportunity to achieve pregnancy.

## Figures and Tables

**Table 1:** Motile sperm concentration, normal morphology, and IUI outcome.

Author	Year	Design (prospective or retrospective)	Number of infertile patients	Number of insemination cycles	IMSC (million)	Normal morphology	IUI outcome	Total motile sperm count*10/6 (before wash)
Ombelet et al.	1997	Retrospective	373	792	<1	<4%	0% CF	
					<1	>4%	7.8%	
					>1	>4%	15%	
Van Voorhis et al.	2001	Retrospective	1,039	3,479			PR 2.3%	<10
							PR 9%	>10
Cohlen et al.	1998	Prospective	74	308	<1		PR 2.6%	
					>1		PR 11.2%	
Wainer et al.	2004	Retrospective	889	2,564	<1	>30%	PR 3.1%	
					2–5		PR 12%	
Dodson & Haney	1991	Retrospective	371	808	<1		CF 0%	
					1–5		CF 17%	
Horvath et al.	1989	Prospective	232	451	<1		CF 2.6%	
					>10		CF 11.6%	
Berg et al.	1997	Retrospective	902	3,037	0.8–1.2		PRcy 8.2%	
					>5		10%	
Miller et al.	2002	Prospective	438	1,114	<10		CF 1%	
					10–20		CF 7.4%	
					>20		CF 12.4%	
Irianni et al.	1993	Retrospective	129	208	>4.7		PR 14%	
Toner et al.	1994	Prospective	126	395	104	9%	PRcy 9%	
Lindheim et al.	1996	Retrospective	42			<4%	PRcy 1%	
						>4%	PRcy 19%	
Montanaro Gauci et al.	1999	Retrospective	273	522		<4%	PRcy 2.6%	
						5–14%	11%	
						>14%	24%	
Shibahara et al.	2004	Prospective	160	682		>15%	PRcy 15%	
Hauser et al.	2001	Prospective	108	264		<4%	4-PRcp 11%	
						14%	36%	
						>14%	50%	
Lee et al.	2002	Prospective	209	244		<4%	PRcy 3.8%	
						4–9%	18.5%	
						>9%	30%	
Dickey et al.	1999	Retrospective	1,841	4,056		<5%	PRcy 0%	
						>5%	11%	
Dickey et al.	1999	Retrospective	1,841	4,056			PRcy 2.3%	<5
							8.5%	>5

CF: Cycle fecundity; IMSC: Inseminated motile sperm count; PR: Overall pregnancy rate; PRcy: Pregnancy rate per cycle; PRcp: Pregnancy rate per couple.

**Table 2:** Sperm preparation methods.

Author	Year	Design (prospective or retrospective)	Number of infertile patients	Number of insemination cycles	Method of preparation	IUI outcome
Carrell et al.	1998	Prospective	363	898	Sperm wash	PR 8.9%
					-Swim-up	14.7%
					-Swim-down	7.7%
					-Percoll density gradient	16.1%
					-Sperm refrigeration/heparin incubation	11%

Dodson et al.	1998	Prospective	80	153	Double cenrifugation	CF 15%
					-Swim-up	14%
					-Percoll density gradient	20%

Morshedi et al.	2003	Prospective	311	676	Simple wash	PR 11.6%
					-Density gradient	14.3%

Lan et al.	2004	Prospective	140	NA	Swim-up	PR 15%
					-Percoll density gradient	20%
					-Wang's tube method	45%

Ren et al.	2004	Prospective	NA	317	Albumin	PR 12.5%
					-Puresperm	7.4%
					-Swim-up	9.6%
					-Percoll density gradient	12.7%

PR: Pregnancy rate; CF: Cycle fecundity; NA: Not available.
